# A Comprehensive Meta-Analysis of MicroRNAs for Predicting Colorectal Cancer

**DOI:** 10.1097/MD.0000000000002738

**Published:** 2016-03-07

**Authors:** Lin Yan, Wenhua Zhao, Haihua Yu, Yansen Wang, Yuanshui Liu, Chao Xie

**Affiliations:** From the Department of Oncology and Pneumology (LY, YW), Shandong Jiaotong Hospital; Department of Oncology (WZ, YL) ; Department of Gastrointestinal Surgery (HY), Shandong Provincial Qianfoshan Hospital, Shandong University; and The Third Department of Internal Medicine (CX), Shandong Tumor Hospital, Jinan, Shandong, China.

## Abstract

Colorectal cancer (CRC) has been defined as a common malignancy due to its prevailing incidence in both males and females. Recently, the intrinsic value of microRNAs (miRNAs) with respect to early cancer diagnosis has been contentious as the diagnostic accuracy of miRNAs significantly varied across different studies. As a result of this, this pioneer meta-analysis was proposed to address this issue.

Qualified studies were obtained through electronic systematical searching in Medline, Embase, and PubMed. On the basis of the random-effects model, we calculated the pooled sensitivity (SEN), specificity (SPE), and area under the receiver operating characteristics curve (AUC) to assess the diagnostic accuracy of miRNAs. Subgroup analysis and meta-regression were implemented to determine how different confounding factors affect the overall diagnostic accuracy which were considered important sources of heterogeneity. All the statistical analyses were conducted with R 3.2.1 software.

We incorporated 103 studies from 36 articles with a total of 3124 CRC patients and 2579 healthy individuals. MiRNAs have a good performance with the following pooled estimates: SEN = 0.769 (95% CI = 0.733–0.802), SPE = 0.806 (95% CI = 0.781–0.829), AUC = 0.857, and partial AUC = 0.773. As suggested by subgroup analyses and meta-regression, multiple miRNAs appeared to be more favorable than single miRNA (AUC: 0.918 > 0.813, partial AUC: 0.848 > 0.701, sensitivity = 0.853 > 0.718, specificity = 0.860 > 0.772). Compared with samples of plasma, blood, tissue, and feces, miRNA obtained from serum samples were more powerful for detecting CRC particularly in Asian.

Our study provided exclusive evidence that multiple miRNAs extracted from serum samples had superior diagnostic performance over single miRNA for screening CRC. Therefore, this approach that is characterized by high specificity and noninvasive nature may assist in early diagnosis of CRC particularly in Asian.

## Abstract 2

Supplemental Digital Content is available in the text

## INTRODUCTION

Colorectal cancer (CRC) that includes colon and rectal cancer is one of the most common malignancies. CRC is ranked as the third highest cancer incidence in males and the second highest cancer incidence in females with 1.2 million annual new cases and over 600,000 annual deaths in the world.^1^ The incidence of CRC varies from region to region; for example, the overall incidence of CRC is significantly higher in Europe, North America, and Oceania compared with South Asia, Central Asia, and Africa.^2^ The present clinical screening method of CRC is mainly based on colonoscopy which is the most reliable screening approach.^3^ However, many patients with CRC are reluctant to undergo colonoscopy screening due to its high operation costs and uncomfortable bowel preparation.^4^ Consequently, an alternative biomarker with high precision and noninvasive nature for CRC detection is urgently needed.

Increasing studies on cancer pathogenesis have shown that both epigenetic alteration and gene mutation could contribute to the malignant transformation of benign adenoma. Furthermore, epigenetic alteration including noncoding RNA alteration, histone modification, and DNA methylation alteration is usually observed in CRC and it may play a role in tumorigenesis.^5^ MicroRNA as a class of noncoding RNA has close relationship with the occurrence and progression of cancer. Many studies have indicated that miRNAs that are characterized by their noninvasive nature can be used as biomarkers for screening, diagnosing, and prognosticating various types of cancer.^6^

MicroRNAs (miRNA) are defined as a kind of small, endogenous, and noncoding RNAs that consist of approximately 20 to 24 nucleotides. MicroRNAs post-transcriptionally regulate gene expression by binding with the 3’-untranslated region of target miRNAs, further contributing to degradation or translational inhibition of mRNA.^7^ In general, miRNAs are first transcribed as long primary transcripts named as pri-miRNA and are processed into precursor miRNAs (pre-miRNA) by enzyme Drosha. Then, pre-miRNAs are transported from cell nucleus into cytoplasm and they receive specific cleave of the enzyme Dicer to transform into double-strands miRNAs. After that, 1 strand of miRNA is degraded and another strand that is the mature miRNA is absorbed into an RNA-induced silencing complex (RISC) to induce gene silencing.^8^ As suggested by the interaction network between miRNAs and mRNAs, 1 miRNA often can target many mRNAs whereas 1 mRNA is usually the target of multiple miRNAs.^9^ MiRNAs are evolutionarily conserved and involved in a variety of critical cellular processes including proliferation, differentiation, senescence, and apoptosis.

It has been reported that significantly differential expression of specific miRNAs was identified between cancer and normal tissues. Therefore, miRNAs as biomarkers can potentially be used for screening, diagnosis, and prognosis of cancer.^10^ However, different miRNAs have been investigated by a large number of studies that affected their comparability with respect to the diagnostic accuracy of CRC. For instance, Ogata-Kawata et al^11^ revealed that miR-23a extracted from serum samples exhibited an unexpectedly high diagnostic accuracy of CRC with 92% sensitivity and 100% specificity. Nevertheless, Luo et al^12^ concluded that miR-92a had a relatively low diagnostic accuracy of CRC with a sensitivity of 68.2% and specificity of 49.4%. Conflicting results due to different miRNA expression profiling, sample source, study subjects, and other uncontrolled factors have impeded the application of miRNAs as a powerful screening and diagnostic tool for cancer. Therefore, this meta-analysis was carried out to investigate whether miRNAs can precisely identify patients with CRC and whether factors such as sample sources and miRNA profiling have significant influence on the diagnostic performance.

## METHODS

Ethics committee is not applicable in this meta-analysis.

### Search Strategy

Online databases including Medline, Embase, and PubMed were searched (updated to June 20, 2015) to identify all articles that evaluated the diagnostic accuracy of miRNAs for CRC. A predefined searching strategy that outlined and combined the following terms was specifically designed for this meta-analysis: (“colorectal tumor” OR “colorectal cancer” OR “colorectal carcinoma” OR “colon cancer” OR “rectal cancer” OR “CRC”) AND (“microRNAs” OR “miRNAs” OR “miR∗”) AND (“diagnoses” OR “diagnostic value” OR “detection” OR “biomarker” OR “sensitivity and specificity” OR “ROC curve” OR “receiver operating characteristics”). The searching strategy was not restricted by any specific languages and relevant reviews or articles on the citation list were independently searched to ensure the completeness.

### Study Selection and Data Extraction

Two reviewers independently screened the titles, abstracts, and full texts of selected articles. Eligible studies included in this meta-analysis were complied with the following criteria: related to the diagnostic value of miRNAs for CRC; gold standard for CRC detection was specified; offered sufficient data to calculate estimates of true positives/false positives and false/true negatives that were used to evaluate the sensitivity, specificity, and sample size. Exclusion criteria were set as follows: publications that were unrelated to the diagnostic value of miRNAs for CRC; studies with duplicated or incomplete data; letters, editorials, commentaries, reviews, or case reports; studies that were not conducted on humans.

Two independent reviewers extracted the following data from eligible studies: the first author, publication year, country, ethnicity, sample size of the case/control group, tumor stages, miRNA profiling, specimen sources, and relevant data for meta-analysis (sensitivity and specificity).

### Statistical Analysis

We performed all statistical analyses using R-3.2.1 software with an additional statistical package of Mada. The random-effects model was used to estimate the pooled sensitivity, specificity, AUC, and partial AUC along with their corresponding 95% confidence intervals (CIs). The sensitivity and specificity of individual studies were plotted to construct the summary receiver operating characteristic curve (SROC) (sensitivity as the vertical axis and 1-specificity as the horizontal axis). Besides that, we calculated the area under the SROC curve (AUC) and partial AUC which are quantitative measurements of the diagnostic accuracy. Moreover, heterogeneity among studies was evaluated by the *χ*^2^ statistics. Potential sources of heterogeneity were investigated using the subgroup analysis and meta-regression.

## RESULTS

### Search Results and Characteristics of Included Studies

**FIGURE 1:**
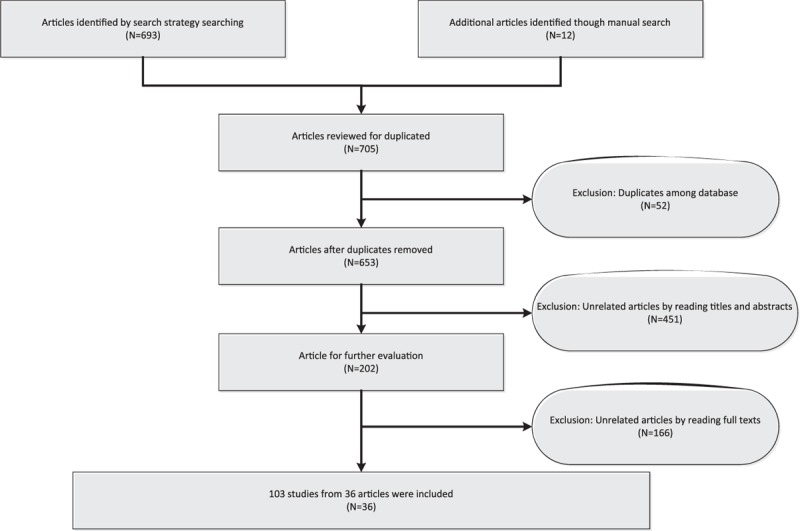
The flow chart of the literature search and selection process.

The flow chart of the entire literature search process is shown in Figure 1. The initial search returned a total of 705 articles (693 from databases, 12 from manual search) of which 52 were excluded for duplicated records. After carefully reviewing titles and abstracts, 451 unrelated articles were excluded and 202 articles were available for further full-text reading. Another full-text reviewed 166 articles were excluded due to the lack of complete data. In the end, there were 36 articles included in this meta-analysis.^11–46^ Table S1 summarized the main characteristics of the 36 included articles ordered by the first author. A total of 103 studies were included in the 36 articles that were published from 2008 to 2015 with a total of 3124 CRC patients and 2579 healthy individuals;.

### Pooled Diagnostic Accuracy of miRNA for Colorectal Cancer

**FIGURE 2:**
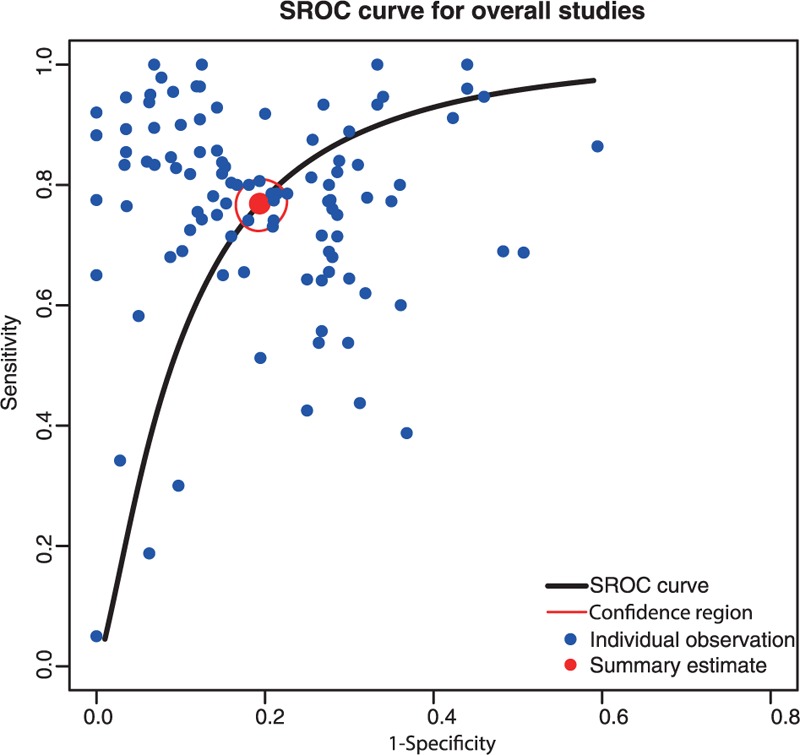
SROC curve for the overall meta-analysis. SROC = summary receiver operating characteristic curve.

The overall predictive accuracy of miRNAs for detecting CRC was pooled from all included studies using the random-effects model and all results are summarized in Table 1. The *χ*^2^ statistic for testing consistent sensitivities and specificities among the included studies were 1263.7 (*P* < 0.01) and 617.3 (*P* < 0.01), respectively, indicating that significant heterogeneity was presented. Sensitivity and specificity forest plots of 5 specimens including serum, plasma, blood, tissue, and feces provided a rough understanding of between-studies heterogeneity. Therefore, we used a random-effects model to estimate the overall diagnostic measurements. The pooled sensitivity and specificity were 0.769 (95% CI: 0.733–0.802) and 0.806 (95% CI: 0.781–0.829), respectively. The overall diagnostic accuracy was further explored by the SROC in which the AUC and partial AUC (0.857, 0.773) was evaluated, further suggesting a relatively high diagnostic accuracy for detecting CRC patients (Figure 2).

**TABLE 1:**
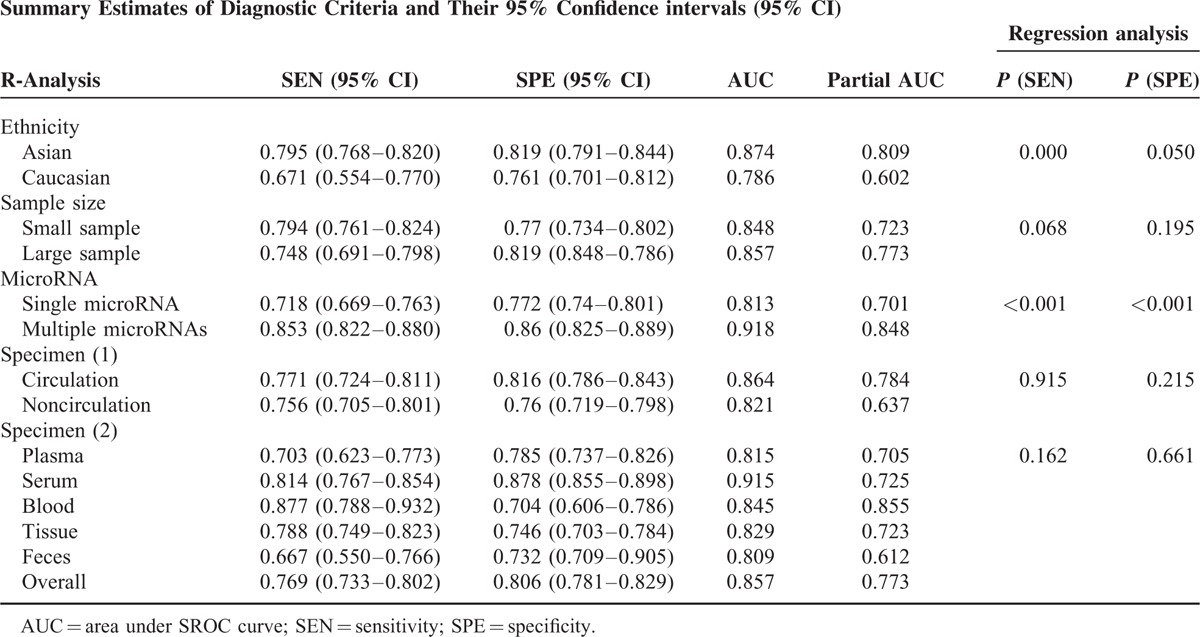
Pooled Results of Diagnostic Accuracy of miRNAs in Diagnosing CRC

### Subgroup and Meta-Regression Analyses

**FIGURE 3:**
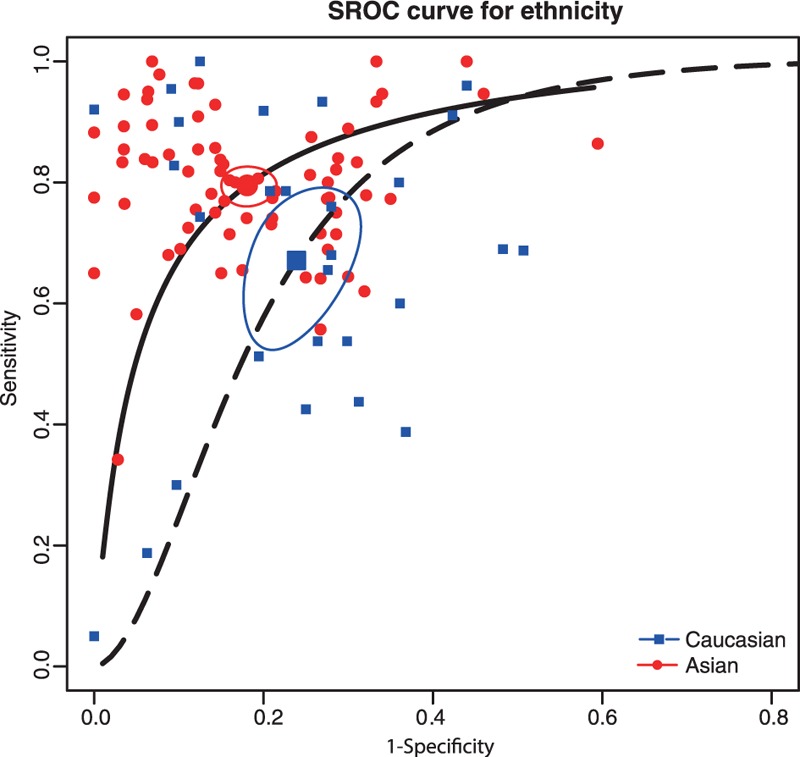
SROC curve for subgroup analysis by ethnicity (Asian vs. Caucasian).

**FIGURE 4:**
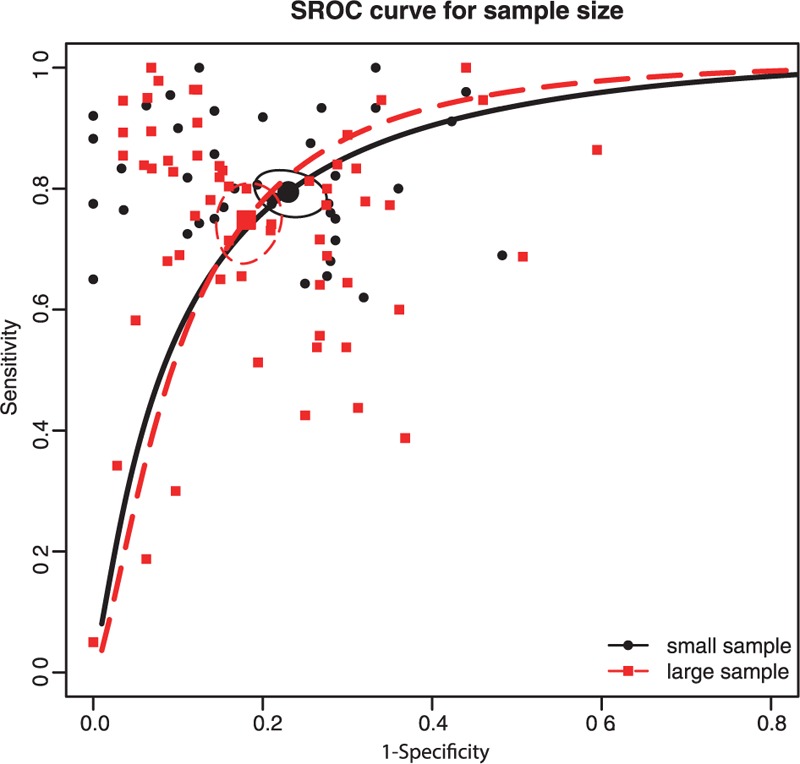
SROC curve for subgroup analysis by sample size (large vs. small).

**FIGURE 5:**
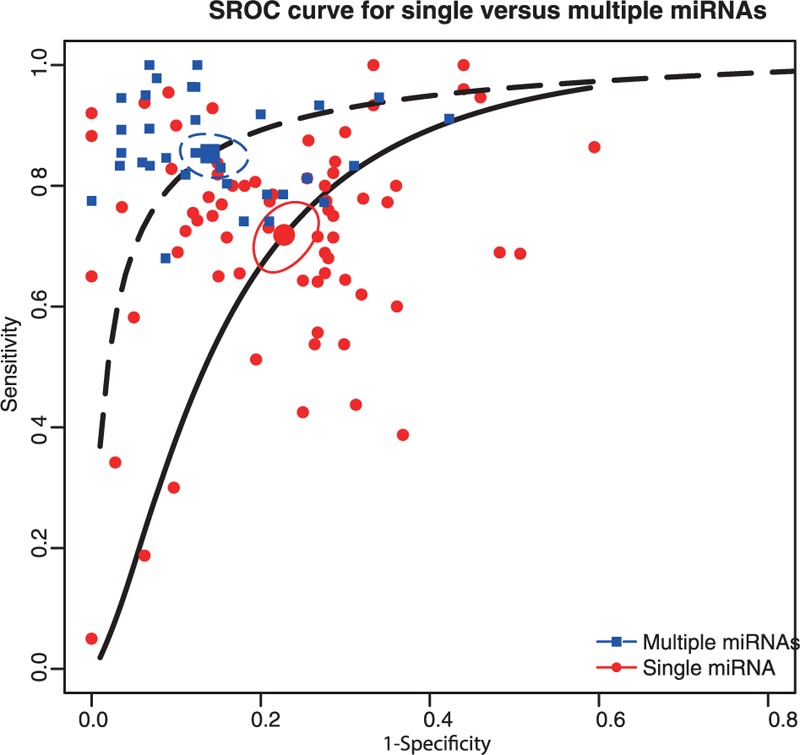
SROC curve for subgroup analysis by single and multiple miRNAs.

**FIGURE 6:**
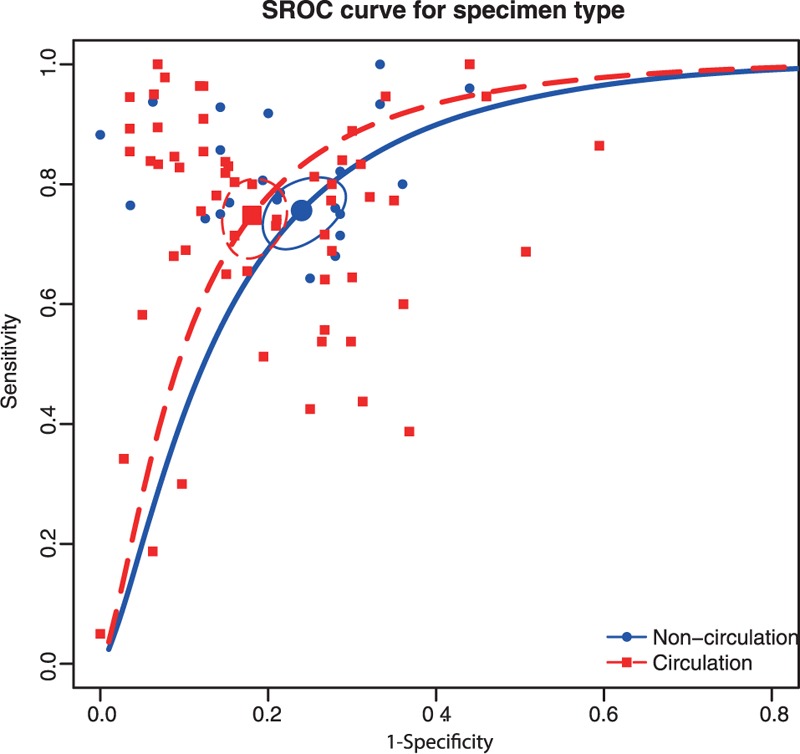
SROC curve for subgroup analysis by specimen type (circulation vs. noncirculation).

In this meta-analysis, we performed subgroup analyses to identify the potential sources of between-study heterogeneity. Table 1 displays pooled estimates including sensitivity, specificity, AUC, and partial AUC for each subgroup. Pooled studies on Asian populations exhibited a higher diagnostic accuracy compared with studies on Caucasian populations, with a sensitivity of 0.795 versus 0.671, specificity of 0.819 versus 0.761, AUC of 0.874 versus 0.786, and partial AUC of 0.809 versus 0.602. The SROC curve by different ethnicity confirmed the above conclusions (Figure 3). Subgroup analysis by sample size revealed that no significant difference in the diagnostic accuracy between studies with large and small sample size, with a sensitivity of 0.748 versus 0.794, specificity of 0.819 versus 0.77, AUC of 0.857 versus 0.848, and partial AUC of 0.773 versus 0.723. This trend was also confirmed by the SROC curve on different sample size (Figure 4). Subgroup analysis by miRNA profiling suggested that multiple miRNAs assays offered more powerful diagnosis of CRC compared with single miRNA assays with a sensitivity of 0.853 versus 0.718, specificity of 0.86 versus 0.772, AUC of 0.918 versus 0.813, and partial AUC of 0.848 versus 0.701. These results were consistent with the pattern exhibited by the SROC curve of miRNAs profiling (Figure 5). In addition, circulating miRNA assays exhibited a higher level of overall accuracy compared with noncirculation miRNA assays, with a sensitivity of 0.771 versus 0.756, specificity of 0.816 versus 0.76, AUC of 0.864 versus 0.821, and partial AUC of 0.784 versus 0.637 (Figure 6). Consequently, we further conducted subgroup analysis by specimen which revealed that serum samples obtained from the circulatory system had higher overall detection accuracy with sensitivity of 0.814, specificity of 0.878, AUC of 0.915 and partial AUC of 0.725 compared with plasma, blood, tissue and feces. Therefore, miRNAs from serum samples are more appropriate than those from other samples with respect to CRC diagnosis.

As suggested by Table 1, results from the meta-regression were consistent with the above conclusions. Ethnicity had significant effect on the pooled sensitivity (*P* < 0.01), while such effect was not significant on the pooled in specificity (*P* = 0.05). Moreover, sample size did not significantly affect the pooled sensitivity (*P* = 0.068) or specificity (*P* = 0.195) which was strongly consistent with the conclusion provided by the subgroup analysis of sample size. Meta-regression also suggested that the individual sensitivity (*P* = 0.915, 0.162) and specificity (*P* = 0.215, 0.661) did not significant vary with different specimen. However, MicroRNA profiling (single, multiple) significantly affected the sensitivity (*P* < 0.01) and specificity (*P* < 0.01) of diagnostic accuracy provided by individual studies. Therefore, meta-regression enabled us to identify that both ethnicity (*P* < 0.01) and miRNA profiling (*P* < 0.01) may contribute to the heterogeneity of the diagnostic accuracy among the included studies.

## DISCUSSION

Epigenetic alteration including differential miRNA expression is one of most important factors for CRC occurrence and progression.^5^ Recent studies have shown that some classes of miRNAs can behave as biomarkers for CRC diagnosis.^6^ Advantages of miRNAs as useful biomarkers include accurate diagnostic value, stable existence in human bodies, and noninvasive nature in the process of detection. However, conflicting results have appeared in studies on different miRNAs for screening and diagnosing CRC. Consequently, we performed this meta-analysis to address this issue and verify the intrinsic diagnostic value of miRNAs for CRC detection.

Our study revealed that miRNAs can exert relatively high screening and diagnosis accuracy for CRC with an overall sensitivity of 0.769, specificity of 0.806, and AUC of 0.857. In addition, subgroup analyses have identified ethnicity, miRNA profiling, and specimen types as potential sources of heterogeneity. For instance, miRNAs provided more precise diagnosis of CRC in Asian compared with that in Caucasian. Besides, multiple miRNAs exhibited higher diagnosis accuracy of CRC than single miRNA. Therefore, it appeared that using multiple miRNAs for detecting CRC was effective and appropriate in Asian. Furthermore, miRNAs from circulating samples were more powerful than miRNAs from noncirculating samples with respect to CRC detection and serum samples were the most appropriate specimen that can be used for CRC detection.

We discovered that a panel of circulating miRNAs containing 45 members (let-7 g, miR-106a, miR-106b, miR-1246, miR-129-3p, miR-133a, miR-139-3p, miR-143, miR-145, miR-155, miR-15b, miR-17-3p, miR-181b, miR-18a, miR-193a-3p, miR-19a, miR-19a-3p, miR-19b, miR-200c, miR-203, miR-20a, miR-21, miR-221, miR-223-3p, miR-23a, miR-29a, miR-31, miR-331, miR-338-5p, miR-375, miR-378, miR-409-3p, miR-422a, miR-431, miR-532-5p, miR-601, miR-634, miR-7, miR-760, miR-767-3p, miR-877∗, miR-92, miR-92a, miR-92a-3p, miR-93) exhibited an overall diagnosis accuracy with sensitivity of 77.1% and specificity of 81.6%. The detailed information was shown in Table S1;. For instance, circulating microRNA-21 had a moderate sensitivity and specificity (75%, 84%) according to the study conducted by Xu et al, which was consistent with our results (sensitivity: 72%, specificity: 80%). Hence, the above evidence revealed that circulating microRNA-21 can be regarded as a novel diagnostic biomarker for CRC.

This is the first time to report the overall diagnostic accuracy of miRNAs for CRC and this meta-analysis identified the most appropriate situation in which miRNA was able to provide a precise diagnostic accuracy for CRC. Above all, multiple miRNAs from serum samples could accurately diagnose CRC and this approach is even more reliable in Asian. However, there were some limitations in our study. First, our study only incorporated Asian and Caucasian samples, so the diagnostic performance of miRNAs in other ethnicities remains unknown. Second, although we discovered that multiple miRNAs exhibited more accurate diagnostic value of CRC, this conclusion should be interpreted with discretion as different panels of miRNAs may yield completely different diagnostic results. For instance, Zheng et al^13^ found that a panel of miRNAs including miR-19a-3p, miR-92a-3p, miR-223-3p, and miR-422a had robust detection accuracy with 95% sensitivity and 94% specificity. However, Kanaan et al^33^ suggested that another panel of miRNAs containing miR-31, miR-135b, miR-1, and miR-133a had extremely robust detection accuracy with 100% sensitivity and 85% specificity. This discrepancy may be explained by the fact that different miRNAs played distinctive roles in the formation and progression of CRC. As a result, we suspected that aberrant expressions of certain miRNA that are more correlated with CRC are more likely to be detected than those of other miRNAs. Hence, discovering a panel of miRNAs that are critical to the biological and molecular function of CRC should be prioritized.

In conclusion, our study provided evidence that multiple miRNAs from serum samples exhibited relatively high diagnostic accuracy for CRC and this approach is considered to be more appropriate in Asian. Therefore, miRNAs could serve as a potential biomarker for CRC detection.

## Supplementary Material

Supplemental Digital Content
